# Evaluation of a Novel In Vitro Diagnostic Immunoassay for the Rapid Qualitative Detection of KPC, NDM, OXA-48-like, IMP and VIM Carbapenemases

**DOI:** 10.3390/diagnostics16121818

**Published:** 2026-06-12

**Authors:** Zoe Dunne, Saoussen Oueslati, Hervé Volland, Thierry Naas

**Affiliations:** 1 Faculty of Pharmacy, Royal College of Surgeons (RCSI), University of Medicine and Health Sciences, D02 YN77 Dublin, Ireland; zoedunne22@rcsi.ie; 2 Team ‘Resist’ UMR1184 Immunology of Viral, Auto-Immune, Hematological and Bacterial Diseases (IMVA-HB), INSERM, Faculty of Medicine, Université Paris-Saclay, CEA, 94270 Le Kremlin-Bicêtre, France; amr.project.oueslati.saoussen@gmail.com; 3 Bacteriology-Hygiene Unit, Bicêtre Hospital, Assistance Publique-Hôpitaux de Paris, 94270 Le Kremlin-Bicêtre, France; 4 Médicaments et Technologies Pour la Santé (MTS), Université Paris-Saclay, CEA, INRAE, SPI, 91191 Gif-sur-Yvette, France; herve.volland@cea.fr; 5 French National Reference Center for Antimicrobial Resistance, 94270 Le Kremlin-Bicêtre, France

**Keywords:** LFIA, carbapenemase, detection

## Abstract

**Background/Objectives:** The global spread of carbapenemase-producing Gram-negative bacteria (CP-GNB) represents a major clinical challenge, causing severe hospital-acquired infections with limited treatment options. Accurate and rapid detection is essential for guiding antimicrobial therapy and implementing infection control measures. Lateral flow immunoassays (LFIAs) targeting the five main carbapenemase families are increasingly used in routine diagnostics, and many new commercial assays have recently become available, often without thorough assessment. The continuous evolution of these enzymes under antibiotic pressure requires regular reassessment of assay performance. **Methods:** In this study, we evaluated the Beright Carba-5 assay (Alltest Biotech, Hangzhou, China) targeting the five main carbapenemases (KPC, NDM, OXA-48-like, IMP, and VIM), on a panel of 77 whole-genome sequenced Gram-negative bacterial (GNB) isolates exhibiting reduced susceptibility to carbapenems. Seventy-three were carbapenemase-producing (CP) GNBs, including six VIM-, 18 OXA-48-, 14 KPC-, 9 NDM-, 8 IMP-, 10 multiple carbapenemase-, and eight non-targeted carbapenemase-producers. **Results:** The assay was rapid and easy to use, showing 100% (CI: 73.54% to 100%) specificity, with no false positive results. However, overall sensitivity of CP-GNB detection was lower than expected at 63.08% (CI: 50.20% to 74.72%), with numerous false negatives, particularly among IMP and NDM producers, and to a lesser extent, KPC producers. Detection was more reliable for VIM and OXA-48-like variants. Practical limitations, including insufficient buffer supply, reduced the number of tested isolates from the planned 100 to 77. **Conclusions:** Overall, the Beright assay demonstrated insufficient sensitivity for routine diagnostic use.

## 1. Introduction

Infections caused by multidrug-resistant (MDR) Gram-negative bacteria (GNB) are increasingly reported worldwide and are associated with substantial morbidity and mortality, representing a major public health concern [1]. Rapid identification of these pathogens is essential to guide infection control measures, optimize antimicrobial stewardship, and improve clinical outcomes [2,3,4]. Traditional antimicrobial susceptibility testing often requires 24–48 h or longer, while molecular assays, although considerably faster, are more expensive and often require specialized laboratory equipment [5]. The development of rapid and cost-effective diagnostic approaches is therefore critical.

Among Enterobacterales, the most prevalent carbapenemases are classified according to the Ambler scheme into three groups: class A serine β-lactamases (e.g., KPC), class B metallo-β-lactamases (MBL; e.g., NDM, VIM, and IMP), and class D oxacillinases (e.g., OXA-48-like enzymes) [6,7]. Lateral flow immunoassays (LFIAs) have emerged as valuable tools for antimicrobial resistance detection, enabling rapid (<15 min), simple, and highly accurate identification of the five main carbapenemases [8,9]. These assays can also be applied directly to clinical specimens, including positive blood cultures, urine, and rectal swabs [10].

The NG-Test CARBA 5 (NG Biotech, Guipry, France) was among the first commercially available assays in 2019 [9] and has since been extensively validated, demonstrating excellent diagnostic performance, with sensitivity and specificity approaching those of PCR [11]. Notably, it shows high accuracy for detecting diverse and challenging IMP variants, underscoring its reliability for routine clinical use [11]. Several competitor assays have been commercialized since, with different test performances as compared to NG-Test CARBA 5 [12,13,14,15]. In most cases, NG-Test CARBA 5 has consistently outperformed alternative lateral flow assays; notably, when assessed using well-characterized collections of Enterobacterales isolates, the assay demonstrated high accuracy in the detection of IMP-type carbapenemases [11]. This is of particular importance given the substantial genetic diversity of IMP enzymes, which often complicates their reliable identification.

As LFIAs built to rapidly identify the five major carbapenemase families, including KPC, NDM, VIM, IMP, and OXA-48-like enzymes, become an important tool at the intersection of infection prevention and control (IPC) and antimicrobial stewardship, their biological performances need to be carefully monitored, especially with an evolving epidemiology, with novel variants appearing [16,17], and with the increasingly isolated double or triple carbapenemase producers [18]. From an IPC perspective, detection of any carbapenemase gene should allow implementation of rapid appropriate containment measures—such as contact precautions, patient cohorting, and screening of contacts—without waiting for slower molecular or phenotypic methods [19]. Rapid identification of the specific enzyme type can also inform local epidemiology tracking, helping hospitals detect outbreaks linked to particular carbapenemase families. For antimicrobial stewardship programs, identifying only all carbapenemases present in double or triple producers is mandatory to help clinicians tailor therapy more precisely. Thus, LFIAs must be challenged with the contently evolving epidemiology of carbapenemase-producing Enterobacterales (CPEs) and their test performances need to be regularly and accurately determined [20].

In this study, we evaluated the performance of the Beright Carba-5 assay (Alltest Biotech, Hangzhou, China), a newly developed in vitro diagnostic immunoassay designed for the rapid qualitative detection of the five major carbapenemases on a large panel of carbapenemase variants representing the global epidemiology of carbapenemase producers.

## 2. Materials and Methods

### 2.1. Bacterial Isolates

In this study, we have evaluated 77 whole-genome sequenced Gram-negative bacterial (GNB) isolates exhibiting reduced susceptibility to carbapenems, which were previously described and used in LFIA evaluations [14]. Among these isolates, 73 were carbapenemase-producing (CP) GNBs, including six VIM-, 18 OXA-48-, 14 KPC-, 9 NDM-, 8 IMP-, 10 multiple carbapenemase-, and eight minor carbapenemase-producers (Table 1). In addition, 4 isolates were classified as non-carbapenemase-producing Enterobacterales (non-CPEs). All bacterial isolates were cultured on UriSelect 4 agar plates (Bio-Rad, Marnes-la-Coquette, France) for subsequent testing.

Several variants of the 5 main carbapenemase families were tested either from single- or multiple-carbapenemase producers. These variants have all been isolated in French hospitals and represent the main variants encountered in France and likely in Europe. They included KPC-2, -3, -4, -28, -31, -33, -44, -47, -48, -66, -86, -94, and -178; NDM-1, -2, -4, -5, -6, -7, -9, -14,-24, and -28; VIM-1, -2, -4, -19; IMP-1, -11, -58, -37, -16, and -94; and OXA-48, -162, -181, -204, -232, -244, -370, -405, -484, -505, -519, -517, -515, and -535 (Table 1).

### 2.2. Lateral Flow Immunoassays

**Figure 1:**
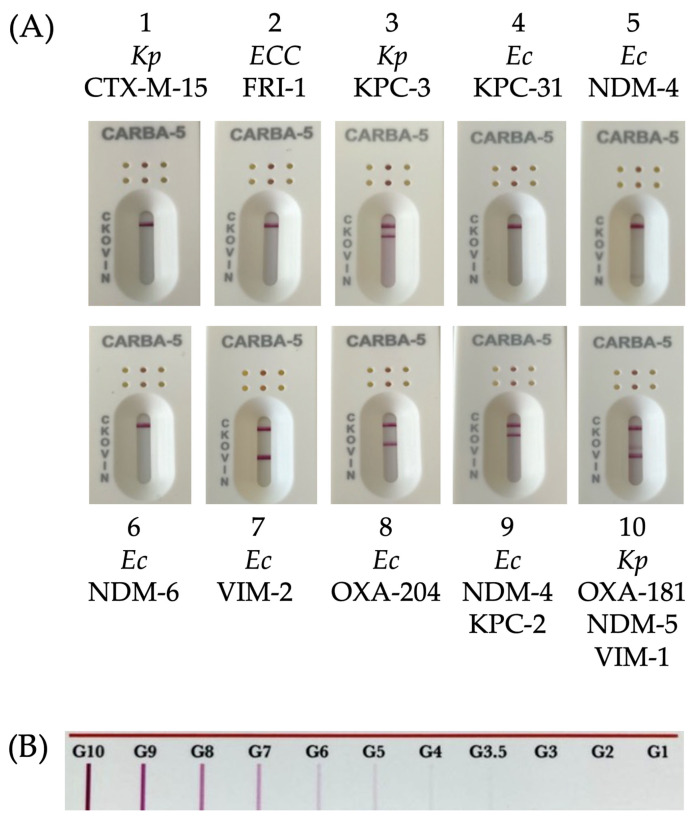
(**A**): Examples of testing results. (1) *K. pneumoniae* CTX-M-15; (2) *E. cloacae* FRI-1; (3) *K. pneumoniae* KPC-3; (4) *E. coli* KPC-31; (5) *E. coli* NDM-4; (6) *E coli* NDM-6; (7) *E. coli* VIM-2; (8) *E. coli* OXA-204; (9) *E. coli* NDM-4+KPC-2; (10) *K. pneumoniae* OXA-181+NDM-5+VIM-1. C: control line; K: KPC; O: OXA-48-like; V: VIM; I: IMP; N: NDM. (**B**) Intensity scale ruler [14].

The Beright Carba-5 assay was used as recommended by the manufacturer (Hangzhou Alltest Biotech, Hangzhou, China). All tests were performed only once, as would be done in a routine clinical diagnostic laboratory when analyzing a bacterial isolate with reduced susceptibility to carbapenems. Briefly, four colonies from a fresh culture of bacteria grown on UriSelect 4 (BioRad, Marne la Coquette, France) were collected using a 1 µL inoculating loop and were thoroughly mixed in 5 drops of the extraction buffer to ensure complete release of the bacterial colonies. After ten minutes of incubation at room temperature, 100 µL was added to the sampling hole of the test cassette. The assay contained separate test lines for each targeted carbapenemase (KPC, NDM, OXA-48-like, IMP, and VIM), along with a control line (Figure 1A). The time to positivity of the test bands and their intensity after the 10 min migration were recorded using a gold color card scale (Figure 1B) [14]. The results were visually assessed after 10 min by two independent observers who were blinded to the identity of the tested isolates. Results appearing after 10 min and those in which the control line failed to appear were considered invalid tests.

In case of discrepant results between Beright CARBA-5 results and the expected result, based on WGS results, a NG-TEST CARBA 5 (NG Biotech) LFIA was used as the gold standard to resolve the problem.

### 2.3. Statistical Analysis

The data were collected and managed with Microsoft Excel 2024. Specificity, sensitivity, and Youden index, along with the 95% Confidence Intervals (95% IC) were calculated using the online MedCalc statistical software version 23.5.9 (https://www.medcalc.org/calc/diagnostic_test.php, accessed on 1 June 2026).

## 3. Results

### 3.1. LFIA Testing Results

The detection rate of single carbapenemase producers was highest for VIM-producing isolates (100%) (Table 1). In contrast, detection of OXA-48 (83.33%), KPC (64.29%) and NDM (22.22%) was variable, while none of the IMP variants were detected. Nine out of the 10 multiple carbapenemase producers were detected as CPEs, but in most cases, only one carbapenemase could be detected. None of the tested bacteria produced another carbapenemase or none (Table 2).

The control line appeared reproducibly after around 60 s with a strong intensity, confirming the good migrations on the strips. The time to positivity of the test lines was variable, going from 2 min for KPC, OXA-48 and VIM, to 4 min for the few NDM positive results, and no positive result was observed for IMP. Similarly, the intensities of the test lines were variable, with usually strong signals for KPC, OXA-48 and VIM enzymes, while for NDM, the signal was very low, and for IMP, no signal was observed (Table 1 and Table 2). The low intensity of test bands made interpretation more difficult (Figure 1A) and reduced the overall reliability of the assay.

For all isolates showing a negative result with the BeRight CARBA-5 assay compared with the expected WGS-based outcome, the results were confirmed using the NG-Test CARBA 5 (NG Biotech). NG-Test CARBA 5 results were fully consistent with WGS data and previous evaluations [14,15]. All carbapenemase variants were detected, except for two KPC variants (KPC-31 and KPC-33), which are known to be missed in previous studies [14].

### 3.2. LFIA Test Performances

The assay demonstrated perfect specificity (100%), with no false positive results obtained throughout (Table 1, Table 2 and Table 3). However, the sensitivities were considerably lower at 63.08% for the detection of bacteria producing at least one of the big five carbapenemases (e.g., 41/65 bacteria). When the detection of an individual carbapenemase is considered, the sensitivity was even lower, 57.89%. A substantial number of false-negative results were observed, particularly among IMP and NDM producers, resulting in reduced overall sensitivity. With double carbapenemase producers, in most cases, only one carbapenemase was detected and NDM was consistently missed.

### 3.3. Practical Evaluation and Limitations

Overall, the assay was easy and rapid to perform. Nevertheless, some practical issues were also encountered during the evaluation. In particular, the volume of the buffer provided with the kits was insufficient to complete all planned experiments. Although the manufacturer indicated that the buffer supplied per kit would be sufficient for testing 100 isolates, only 77 could ultimately be processed. According to the protocol provided, five drops of buffer are required to lyse each bacterial colony prior to application onto the LFIA device. However, contrary to the test protocol, the volume of each drop was closer to 40 µL rather than the reported 25 µL. Consequently, when following the recommended procedure, a shortage of buffer was observed. This shortage not only limited the number of tests that could be performed but also introduced additional logistical constraints during the experimental workflow.

Testing 100 well-characterized Gram-negative isolates also remains limited when considering the extensive diversity of carbapenemase variants described in reference databases such as the Beta-Lactamase DataBase (BLDB; http://www.bldb.eu [7]). As of February 2026, more than 68, 284, 84, 95, and 111 variants of OXA-48, KPC, NDM, VIM, and IMP, respectively, have been reported. In this study, only 77 isolates could be evaluated, including those producing 14 (20%) OXA-48-, 13 (4.6%) KPC-, 10 (12%) NDM-, 5 (5.3%) VIM-, and 6 (5.4%) IMP-variants, which represents an important limitation in terms of capturing the full molecular diversity of carbapenemases.

However, it is unlikely that testing the additional 23 isolates would have substantially altered the overall conclusions, as the selected panel of 77 isolates already reflected the most prevalent carbapenemases encountered in France. Moreover, this collection is likely broadly representative of global epidemiology, since approximately half of the isolates originated from imported cases spanning diverse geographic regions, thereby providing a realistic overview of circulating carbapenemase diversity in clinical settings.

## 4. Discussion

Effective control of carbapenemase-producing Gram-negative bacteria (CP-GNB) relies on integrated strategies, including enhanced surveillance, antimicrobial stewardship, and strict infection prevention measures. These efforts are critically dependent on the availability of rapid and reliable diagnostic tools to limit transmission and improve patient outcomes [3,21,22]. LFIAs represent valuable tools for antimicrobial resistance detection, offering substantial clinical advantages [8,20]. They are simple to perform, require no specialized training, and provide results within 15 min [8,11,20]. Moreover, LFIAs demonstrate high diagnostic accuracy, with near-complete sensitivity and specificity for the detection of the five main carbapenemases in cultured Gram-negative bacteria [11]. Their applicability to direct testing of clinical specimens further supports timely identification of resistant pathogens, thereby facilitating prompt infection control interventions and appropriate antimicrobial therapy [2,10]. Finally, their ease of use, the storage at room temperature and the standalone test (no equipment) make them suitable for laboratories with limited resources, including those in low-income countries. These features align well with the World Health Organization ASSURED criteria (Affordable, Sensitive, Specific, User-friendly, Rapid, Equipment-free, Deliverable) [8,23]. However, the continuous emergence of novel carbapenemase variants, driven by antibiotic selective pressure, remains a significant challenge. Therefore, continuous monitoring of test performances of existing or novel LFIA assays is mandatory for their safe clinical use.

The Bright Carba-5 assay, which is commercially available, is reported in the manufacturer’s leaflet to have promising performance, with 96% sensitivity and 99% specificity based on 200 tested isolates. However, no details are provided regarding the bacterial species included or the specific carbapenemase variants evaluated. In our study, we assessed a diverse collection of whole-genome sequenced bacterial species expressing the most common “big five” carbapenemase variants. The performance observed was markedly lower than that reported by the manufacturer, with only 63% detection of CPEs and 58% correct identification of carbapenemases. In comparison, the NG-Test CARBA 5 (NG Biotech) has been extensively validated worldwide and, as recently shown in a meta-analysis assessing its clinical performance for the detection of carbapenemase-producing Gram-negative bacteria, demonstrates excellent diagnostic accuracy, with a sensitivity and specificity of 0.97 (95% CI: 0.97–0.98) and 0.99 (95% CI: 0.99–1.00), respectively, approaching that of PCR [11]. It demonstrates high accuracy across diverse and challenging variants, supporting its reliability for routine clinical use [11]. Since its introduction, several competing assays have been commercialized with variable performance compared to NG-Test CARBA 5 [12,13,14,15]. Overall, NG-Test CARBA 5 has consistently outperformed alternative LFIAs, including in studies using well-characterized Enterobacterales collections, where it showed particularly strong detection of IMP-type carbapenemases [11].

The Beright Carba-5 assay failed to detect five out of 14 KPC variants, namely KPC-31, -33, -178, -48, and -66. Whether these variants should be detected remains debatable, as they are associated with resistance to ceftazidime–avibactam (CAZ/AVI) and have largely lost their carbapenemase hydrolytic activity, effectively behaving as extended-spectrum β-lactamases (ESBLs). As a result, infections caused by Enterobacterales producing these variants may still be effectively treated with carbapenems [24]. This issue is particularly relevant in the context of rapid diagnostic tests, including LFIA, which are increasingly used to identify carbapenemases and enable early initiation of appropriate therapy in bloodstream infections [25,26,27]. While CAZ/AVI is recommended as a first-line treatment for infections caused by KPC-producing organisms, the emergence of variants that confer CAZ/AVI resistance while restoring carbapenem susceptibility complicates therapeutic decision-making. Detection of such variants (e.g., KPC-31 and -33) could lead to inappropriate use of CAZ/AVI, resulting in therapeutic failure and potential selection of resistance in other members of the gut microbiota. In contrast, because these variants no longer function as true carbapenemases and may remain susceptible to carbapenems, they could theoretically be treated with carbapenem-based regimens [24].

We also observed poor detection of NDM, with seven of nine variants missed (namely NDM-1, -2, -5, -6, -9, -14, and -24), as well as a complete absence of IMP variant detection. This is particularly worrisome, as NDM-like MBLs are becoming increasingly important worldwide due to their rapid global dissemination and their ability to confer resistance to almost all β-lactams, including carbapenems, the limited therapeutic options and their frequent association with multidrug-resistant and high-risk clones [28]. In contrast, IMP-type carbapenemases, even though rare in Europe, are highly prevalent in several parts of Southeast Asia, where they are among the most frequently reported MBLs in clinical Enterobacterales and non-fermenters [28]. Their endemic circulation in this region contributes significantly to the local burden of carbapenem resistance. IMP enzymes are known to be particularly challenging to detect due to their substantial genetic diversity, which often compromises reliable identification [11,14,15]. In contrast, OXA-48 detection showed comparatively better performance, with only three variants missed, namely OXA-232, OXA-515, and OXA-535, out of the 18 tested.

The emergence and dissemination of double or triple carbapenemase-producing Enterobacterales (CPE) is an increasing global concern, as isolates co-harboring multiple carbapenemase genes combine different resistance mechanisms and significantly reduce therapeutic options [18]. Their spread complicates both infection control efforts and diagnostic accuracy, while also undermining the effectiveness of rapid tests and empiric antimicrobial strategies [29]. All multiple carbapenemase-producing isolates were correctly identified as CPEs, except for one *K. pneumoniae* isolate producing NDM-5 and OXA-232, which was missed. However, in most cases, only a single carbapenemase was detected, even when multiple enzymes were present. This limitation is particularly concerning when such assays are intended for use in antimicrobial stewardship programs, where the identification of a specific carbapenemase directly guides targeted antimicrobial therapy [30]. As LFIAs are now widely implemented for carbapenemase detection in bloodstream infections, a high level of analytical accuracy is essential [31]. False-negative results may lead to inappropriate antimicrobial choices, resulting in therapeutic failure and potentially promoting the selection of resistance within the gut microbiota. For example, detection of OXA-48 enzymes may support the use of β-lactam/β-lactamase inhibitor combinations such as ceftazidime–avibactam, whereas the additional presence of MBLs such as NDM or VIM would necessitate alternative therapeutic strategies, including aztreonam-based combinations.

## 5. Conclusions

LFIA has been shown to bridge a critical diagnostic gap by combining the speed of point-of-care testing with clinically actionable resistance information. This facilitates earlier IPC interventions and supports more judicious antimicrobial use, both of which are essential for limiting the spread of multidrug-resistant organisms [11]. However, for novel LFIA assays to be used safely in clinical decision-making, they must demonstrate a high level of accuracy and reliability.

In this study, the BeRight Carba-5 immunochromatographic assay was evaluated for rapid detection of the five major carbapenemases (KPC, NDM, VIM, IMP, and OXA-48-like). Although the assay was rapid and easy to use, its overall performance was limited due to frequent false-negative results, particularly among NDM- and IMP-producing isolates. Detection of VIM and OXA-48-like enzymes was more reliable, but overall sensitivity remained low (63%), far below the 96% reported by the manufacturer. Consequently, the assay is not suitable as a standalone confirmatory test for carbapenemase-producing Enterobacterales. Performance was further reduced in isolates carrying multiple carbapenemases, with only 58% correctly identified.

Our findings indicate that the assay lacks sufficient diagnostic accuracy for confirmatory CPE testing in clinical microbiology laboratories and should not be integrated into antimicrobial stewardship programs, as nearly 42% of carbapenemases may remain undetected, increasing the risk of inappropriate clinical decisions.

## Figures and Tables

**Table 1:** Detection of single carbapenemase producers by the Beright Carba-5 assay.

Mechanisms of Resistance No. of Isolates = 67	Species	Result	Time in Seconds (Intensity at 10 min) ^a^
Non-carbapenemase producers (*n* = 4) Case + impermeability (2); CTX-M (*n* = 1); ACC-1 (*n* = 1)	*E. coli* (*n* = 2); *K. pneumoniae* (*n* = 1); *E. cloacae* complex (*n* = 1)	Negative	C: 39 +/− 9 ^b^
Minor carbapenamases (*n* = 8) Sme-2, IMI-1, GES-5, FRI-1, OXA-23, OXA-372, TMB-1, GIM-1	*E. cloacae* complex (*n* = 5), *C. freundii* (*n* = 1), *S. marcescens* (*n* = 1), *P. mirabilis* (*n* = 1)	Negative	C: 51 +/− 19
KPC-type (*n* = 14) KPC-2 (3), -3 (2), -31, -33, -44, -47, -48, -66, -86, -94, -178	*E. coli* (*n* = 5), *S. marcescens* (*n* = 1), *K. pneumoniae* (*n* = 8)	9 detected/14 (64.29%) not detected KPC-31, -33, -48, -66, -178	T: 94 +/− 51 ^b^ (G5-10)
NDM-type (*n* = 9) NDM-1, -2,-4, -5, -6, -7, -9, -14,-24	*E. coli* (*n* = 7), *K. pneumoniae* (*n* = 1), *A. baumannii* (*n* = 1)	2 detected/9 (22.22%) not detected NDM-1, -2, -5, -6, -9, -14, -24	T: 247 +/− 178 (G4-5)
VIM-type (*n* = 6); VIM-1 (2), -2, -4 (2), -19	*K. pneumoniae* (*n* = 2), *E. cloacae* complex (*n* = 2), *E. coli* (*n* = 2)	6 detected/6 (100%)	T: 125 +/− 84 (G6-10)
IMP-type (*n* = 8) IMP-1 (3), -11, -58, -37, -16, -94	*E. coli* (*n* = 1), *K. pneumoniae* (*n* = 1), *S. marcescens* (*n* = 1), *C. freundii* (*n* = 1), *Acinetobacter* sp. (*n* = 2), *Pseudomonas* sp. (*n* = 2)	0 detected/8 (0%) None detected	C: 63 +/− 19
OXA-48 (*n* = 18) OXA-48, -162, -181 (2), -204 (3), -232, -244 (3), -370, -405, -484, -515, -517, -519, -535	*K. pneumoniae* (*n* = 6), *E. coli* (*n* = 9), *Shewanella* sp. (*n* = 1), *Shewanella bicestrii* (*n* = 1), *S. marcescens* (*n* = 1)	15 detected/18 (83.33%) not detected OXA-232, -515, -535	T: 139 +/− 102 (G4-10)

^a^: Time to visible band in seconds and intensity of band after 10 min of migration, as compared to intensity ruler, Figure 1B. ^b^: C: control line; T: test line.

**Table 2:** Detection of multiple carbapenemase producers by the Beright Carba-5 assay.

Mechanisms of Resistance N° of Isolates = 10 N° of Carbapenemases = 21	Species	Result	Time in Seconds (Intensity at 10 min) ^a,b^
Multiple Carbapenamase Producers (*n* = 10)		9 CPE detected/10 (90%)	
Carbapenamases tested NDM-1 + OXA-48 NDM-5 + OXA-232 NDM-1 + VIM-2 OXA-181 + NDM-5 NDM-7 + KPC-4 NDM-4 + KPC-2 KPC-28 + OXA-48 OXA-181 + NDM-5 + VIM-1 OXA-505 + VIM-1 VIM-4 + OXA-48	*E. coli* *K. pneumoniae* *E. coli* *E. coli* *E. cloacae* complex *K. pneumoniae* *E. coli* *K. pneumoniae* *C. freundii* *E. cloacae* complex	12 carbapenemases detected/21 (57%) NDM-1 not detected NDM-5 + OXA-232 not detected not detected NDM-1 NDM-5 not detected NDM-7 not detected NDM-4 not detected KPC-28 not detected NDM-5 not detected	T KPC: 110 +/− 45 (KPC: G5-10) T VIM: 167 +/− 135 (VIM: G6-10) T OXA: 122 +/− 90 (OXA: G3.5-10)

^a^: Time to visible band in seconds and intensity of band after 10 min of migration, as compared to intensity ruler, Figure 1B. ^b^: C: control line; T: test line.

**Table 3:** Overall test performances.

	Carbapenemase Producer Detected	Carbapenemase Detected
Specificity	100% CI (73.54% to 100%)	100% CI (73.54% to 100%)
Sensitivity	41/65 = 63.08% CI (50.20% to 74.72%)	44/76 = 57.89% CI (46.02% to 69.14%)
Youden’s index	0.63	0.58

Sensitivities and specificities were calculated with their respective 95% confidence intervals using MedCalc (https://www.medcalc.org/en/calc/diagnostic_test.php, accessed on 1 June 2026). Youden’s index (sensitivity (%) + specificity (%) − 100) calculates the efficiency of the immunoassay. A score of 100 indicates a perfect test [2]. Disease prevalence of 1%, corresponding to the French prevalence for CPEs in blood cultures, was used to calculate.

## Data Availability

The original contributions presented in this study are included in the article. Further inquiries can be directed to the corresponding author.
